# Development of a Novel DGT Passive Sampler for Measuring Cs-137 In Situ in Marine Environments

**DOI:** 10.1021/acs.analchem.3c03767

**Published:** 2024-02-13

**Authors:** Ahmed Elsenbawy, Jacqueline M. Pates, Nariman H. M. Kamel, Tarek Morsi, Mohammed Mekewi, Ayman El-gamal, Nabawia A. Moussa, Hao Zhang

**Affiliations:** † Radiation Protection Department, Nuclear Research Center, Egyptian Atomic Energy Authority, Cairo 13759, Egypt; ‡ Lancaster Environment Centre, Lancaster University, Lancaster LA1 4YQ, United Kingdom; § Department of Chemistry, Faculty of Science, Ain Shams University, Cairo 11566, Egypt; ∥ Marine Geology Department, Coastal Research Institute, National Water Research Center, 15, St. Elpharanaa, Elshalalat, Alexandria 21514, Egypt

## Abstract

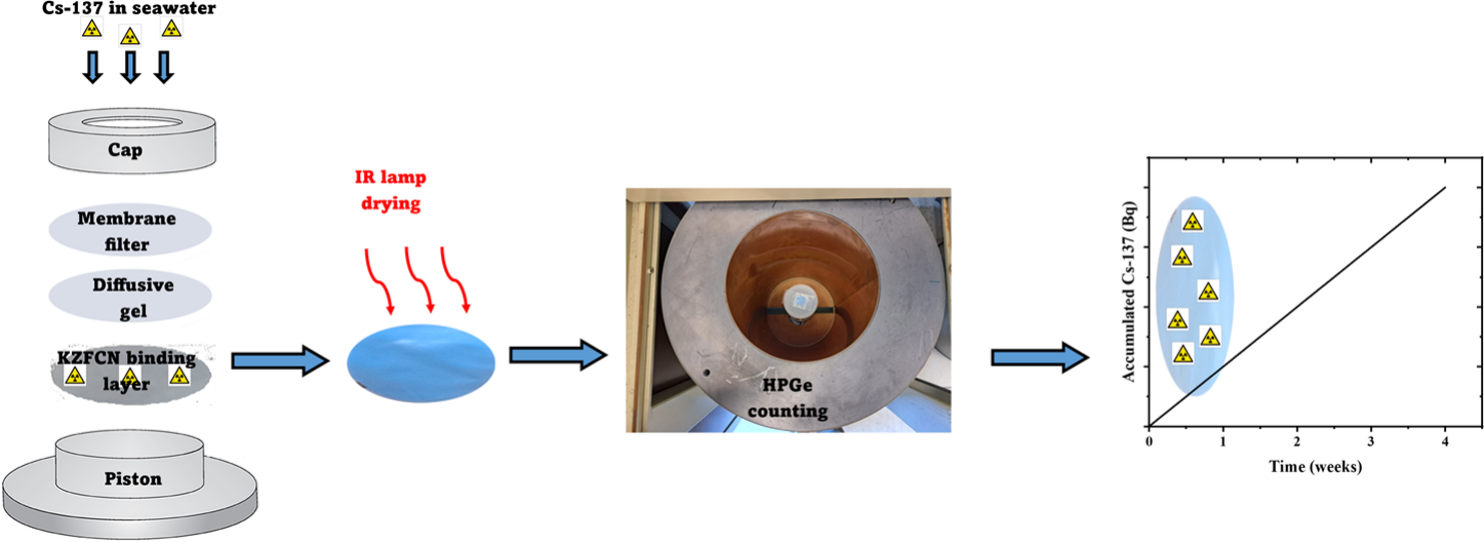

Cs-137 is the most released fission product in the marine environment. It is important to develop a robust in situ technique for its monitoring. The existing diffusive gradients in thin films (DGT) passive sampling techniques for in situ measurement of Cs^+^ have some limitations due to the ion competition and high pH of seawater. A new DGT sampler based on potassium zinc hexacyanoferrate (KZFCN) as a binding layer has been developed and investigated for the measurement of the time-weighted average concentration of Cs-137 in seawater. This binding layer proved a working pH range of 2–12 and an ionic strength of up to 0.75 M. Two types of diffusive gels were tested and agarose gel (AGE) was chosen for the KZFCN-DGT sampler. The measured Cs^+^ diffusion coefficient (1.71 × 10^–5^ cm^2^·s^–1^ at 25 °C) in the diffusive gel from seawater was within the expected range published in the literature. The measured concentrations of Cs-137 in seawater obtained by laboratory deployments of the KZFCN-DGT samplers for up to 4 weeks showed good precision (RSD = 13%) and accuracy (relative error = 8.5%) values. The performance test results demonstrated that the KZFCN-DGT sampler is suitable for long-term monitoring of Cs-137 in seawater due to its high capacity and resistance to ion competition and high pH.

The global emerging need for zero-carbon-emitting energy sources has raised the demand for nuclear fission energy as cleaner intense resources. However, nuclear accidents, controlled releases of the backend nuclear fuel cycle, and weapon testing have been releasing considerable amounts of anthropogenic radionuclides to the marine environment. Among them, Cs-137 which is a key fission product radionuclide is the most released. The accident occurred at TEPCO Fukushima Dai-ichi Nuclear Power Plant in 2011 released an estimated amount of 2.3–6 PBq^1^ of Cs-137 to the Pacific Ocean. Nuclear fuel reprocessing facilities at Sellafield (UK) have released considerable quantities of Cs-137 for decades into the Irish Sea.^2^ The ecological half-life of Cs-137 in the marine ecosystem is variable ranging from a few days^3^ to 14 years.^4^ Its biochemical similarity with potassium increases its bioavailability. Although the activity concentration of Cs-137 in the seawater is within the range of few Becquerels per cubic meter^5^ except for contaminated locations, bioaccumulation and magnification at the higher level of the marine food chain increase the Cs-137 activity concentration above the permissible levels in some species,^6,7^ which are consumed by humans. Consequently, monitoring programs and research studies have been giving priority for Cs-137 activity concentrations, trends, and concentration factors in contaminated areas. For example, Cs-137 analysis and accumulation monitoring in edible fishes and marine wildlife have been given great attention in Japan,^6^ the European Union,^7,8^ and U.K.^9^ Regulators and stakeholders make use of concentration factors and the derived concentration guideline levels for calculations of regulated discharges. Therefore, Cs-137 monitoring in seawater is important for decision-making and for supporting disposal programs.

The direct measurement of Cs-137 in seawater samples using a high-resolution HPGe γ spectrometer is not feasible because its activity concentration is below the detection limit. The current adopted method in most laboratories involves the collection of large volumes of samples ranging from 10 to 200 L, followed by acidification for sample preservation. After that, the sample is preconcentrated by the sorption on ammonium molybdophosphate (AMP)^10^ or transition metal ferrocyanides.^11^ Finally, the settled solid is filtered, dried, and then counted using the HPGe γ spectrometer. The drawbacks of this method include the high cost of sampling and transporting large volumes of water, the susceptibility of the sample to changes during storage and transportation, the elaborate sample preparation, and the unrepresentativeness of the bulk sampling. Recent developments in passive sampling techniques have offered opportunities to overcome those problems.

The diffusive gradients in thin films (DGT) technique is a passive sampling and chemical speciation technique developed by Davison and Zhang,^12^ which measures time-weighted averaged concentrations of metals and radionuclides. DGT techniques were developed for the measurement of cesium in aquatic environments. Chang et al.^13^ investigated the use of the DGT technique based on a cation exchange resin (AG50W-X8) gel for Cs^+^ measurements. Its response was linear only in soft water for up to 20 h deployment due to capacity limitation and competition with other ions. After that, Murdock et al.^14^ developed a DGT technique with AMP immobilized gel for Cs-137 measurement. It was successful in field measurements in Lake Llyn Trawsfynydd for up to 1 month. But, further deployments were associated with an increasing error due to the degradation of AMP. Later, Gorny et al.^15^ further investigated the DGT performance and stability of this binding phase. They found that it was not stable at pH >6. Its deployment time was limited by ion competition at high levels of Na^+^, Ca^2+^, and Mg^2+^. Li et al.^16^ assessed a DGT sampler with copper ferrocyanide (CFCN) immobilized gel as a binding layer for the measurement of cesium in river water. The uptake was linear and agreed with the theoretical calculations for up to 2 weeks but never tested in seawater. However, its working pH range of 4–8 suggests that it is not suitable for seawater applications. The most recent publication concluded that a new binding phase for Cs^+^, whose properties are not affected by environmental conditions, is still a gap that needs to be fulfilled.^15^

Potassium zinc hexacyanoferrate (KZFCN) is a selective sorbent for Cs^+^, which exhibits a wide working pH range^17^ compared to other sorbents. Because of its fast kinetics for the removal of Cs^+^, it was used as a cellulose composite in the Soviet Union as a rapid method for the preconcentration of Cs-137 from surface waters and even seawater.^18^ Yasutaka^19^ used a nonwoven fabric loaded with KZFCN as a cartridge for the preconcentration of Cs-137 from natural waters. High recovery was achieved in the pH range of 3–9. In spite of the better characteristics of KZFCN in retaining Cs-137 under severe conditions of pH and ionic strength and decomposition resistance, it has never been used as a DGT binding phase. In this study, a new binding gel based on KZFCN was reported as a Cs-137-selective DGT binding phase for long-term deployments in seawater. DGT samplers and the binding gel were tested in the laboratory at different solution conditions to assess the DGT performance for Cs-137 measurement. This approach was evaluated for long-term deployment in artificial seawater spiked with Cs-137.

## Materials and Methods

### Materials

All solutions were prepared by using Milli-Q water. Polypropylene containers and DGT holders were acid-cleaned in 10% HNO_3_ solution for 24 h and then rinsed with double distilled water prior to use. The following chemicals were used: Agarose (Merck), acrylamide (Fisher Scientific), bis(acrylamide) (Merck), zinc acetate decahydrate (Merck), K_4_[Fe(CN)_6_]·3H_2_O (Merck), NaCl (Merck), NaNO_3_ (Merck), MgCl_2_ (Merk), CaCl_2_ (Merck), agarose derivative cross-linker (DGT Research Ltd.), ammonium persulfate (Merck), tetramethylethylenediamine (Merck), and polyethersulfone membrane filters (Pall, 0.45 μm pore size).

### Gel Preparation

Agarose (AGE) and agarose cross-linked polyacrylamide (APA) diffusive gels and bis(acrylamide) cross-linked acrylamide (BPA) gel were prepared as previously mentioned in other references.^15,20^ The diffusive gels were stored in 0.01 M NaNO_3_ in a fridge. The binding phase was prepared by immobilizing KZFCN in a BPA or APA gel using the following procedure. A gel sheet of 0.4 mm thickness was placed in a 200 mL solution of 1 M zinc acetate and shaken well for 6 h. Then, it was removed from the zinc acetate solution and briefly rinsed and shaken in a 100 mL solution of K_4_[Fe(CN)_6_] of 0.5 M solution for 6 h. The gel turned white due to the formation of zinc hexacyanoferrate within it. Finally, the gel was retrieved from the solution, rinsed, and shaken in Milli-Q water for 10 h at least. Water was changed at least 3 times to remove excess reagents from the gel sheets. KZFCN binding gel disks were stored in a 0.01 M NaNO_3_ solution in a fridge and used within a month.

### Investigation of the Uptake Kinetics, Elution Efficiency, and Reactivity of the Binding Phase

All of the experiments were undertaken using a working solution of carrier-free Cs-137 in 0.01 M NaNO_3_ at an activity concentration of 6.1 Bq·mL^–1^, unless otherwise mentioned. The binding gel uptake kinetics for Cs-137 were assessed by gently shaking gel disks in 20 mL aliquots of the working solution for specific time intervals (2, 4, 6, 8, 10, and 20 min). The gels were immediately removed after shaking, and the solutions were measured for the remaining Cs-137 activity concentration. The effect of pH on the binding properties of the gel disks was studied in the range of 2–12. The pH values of 20 mL portions of the working solution were adjusted using NaOH and HNO_3_ solutions. Binding gel disks were equilibrated in them by shaking for 24 h and then removed. The solution portions were measured for Cs-137 activity concentration remaining after equilibration. The influence of ionic strength on the binding gel uptake was investigated in the range of 0.01–1.5 M NaNO_3_. The NaNO_3_ concentrations in 20 mL aliquots of the working solution were adjusted using NaNO_3_ salt. The binding gel disks were shaken with the solutions for 24 h and then eliminated. The solutions were counted for Cs-137 activity after equilibration. The uptake percent (*f*_u_, %) was calculated from the initial activity of the solution (*A*_i_, Bq) and the remaining activity (*A*_f_, Bq) using eq 1:
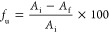
1The elution properties of the KZFCN binding gel were studied using gel disks loaded with known masses of Cs^+^. The loaded disks were immersed in 1 mL of various concentrations of HNO_3_, ammonia, or a mixture of ammonia and ethylenediaminetetraacetic acid for 24 h. The eluates were measured for the Cs^+^ concentration. The elution efficiency (*f*_e_) was calculated from the Cs^+^ masses in the loaded disks (*m*_d_) and the eluates (*m*_e_) using eq 2:
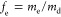
2

### DGT Assemblies

DGT samplers were assembled by laying a KZFCN binding gel on the DGT piston base and a layer of the diffusive gel on the laid binding gel and then covered by a hydrated membrane filter. A piston cap is compressed to hold the layers tightly.

### Diffusion Coefficient Measurement by DGT Deployment Time Series

All DGT experiments were performed in polypropylene containers with a capacity of 3 L at 15 °C, unless otherwise specified. The deployment solutions were allowed to equilibrate with the container, surrounding atmosphere, and temperature. DGT samplers were deployed for specific time intervals in triplicate samplers per interval, and then the binding gels were recovered and measured for the accumulated Cs-137 activity according to the analytical procedures mentioned later. Prior to the deployment and after retrieval of the samplers, aliquots of the deployment solution were taken and measured for the Cs-137 activity concentration. For diffusion coefficient (*D*) measurements, KZFCN-DGT samplers were deployed in 3 L of 1.93 Bq·mL^–1^ Cs-137 in 0.01 M NaNO_3_ in a time series. The samplers were lifted from the solution after specific time intervals (2, 4, 6, 12, 18, and 24 h). In another experiment which investigated D of Cs^+^ in artificial seawater, KZFCN-DGT samplers assembled with AGE diffusive gel were deployed for time intervals of (2, 4, 6, 12, 18, and 24 h) in artificial seawater solution prepared according to the method mentioned in the Supporting Information at pH 8.5 and spiked with 2.4 Bq·mL^–1^ Cs-137. The diffusion coefficient (*D*, cm^2^ s^–1^) was calculated from the accumulated activity (*M*, Bq), the solution activity concentration (*C*_soln_), the exposure area (*A*, cm^2^), time of deployment (*t*, s), and the diffusive layer thickness (Δ*g*, cm) using eq 3:
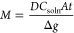
3

The slope of a linear plot of *M* vs *t* was used to calculate *D* according to eq 4:
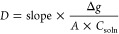
4

The measured *D* at a certain temperature was corrected to 25 °C using the Stokes–Einstein equation given in eq 5:
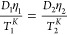
5where (η, mPa·s) is the water viscosity and (*T^K^*, °K) is the absolute temperature.^20^

### Effect of Solution pH, Ionic Strength, and Interfering Ions on the DGT Performance

To investigate the effect of the solution components: pH, ionic strength, and interfering ions on the DGT samplers’ performance, they were deployed in solutions of varying concentrations and values of the parameters for 24 h. For the assessment of the effect of pH on the DGT performance, samplers were deployed in 0.01 M NaNO_3_ solutions of pH 2–12 spiked with 2.4 Bq·mL^–1^ Cs-137. The ionic strength effect was investigated in solutions at a range of NaNO_3_ concentration of 0.01–0.75 M spiked with 2.4 Bq·mL^–1^ Cs-137. The influence of interfering ions was investigated in solutions of 2.4 Bq·mL Cs-137 in 300 mg·L^–1^ Ca^2+^, Mg^2+^, or K^+^. The time-weighted average concentration (*C*_DGT_, Bq·mL^–1^) was calculated by eq 6
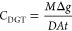
6

### Diffusive Boundary Layer Measurement

The diffusive boundary layer (DBL) is a stagnant water layer that may exist next to the material diffusive layer, which increases the ion diffusion path and decreases the accumulated mass over time. This layer exists even in a vigorously stirred solution.^21^ The DBL thickness (δ_DBL_) increases when the solution stirring is not optimal. δ_DBL_ was measured by deploying DGT samplers equipped with a cap of 2.54 cm^2^ exposure area and different AGE diffusive gel thicknesses (δ_mdl_) for 11h in the same artificial seawater solution container used in the *D* measurement by time series section and spiked with 2.4 Bq·mL^–1^ Cs-137, and the measurements were then calculated using eq 7:
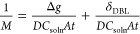
7

δ_DBL_ was calculated by dividing the intercept by the slope of a linear plot of Δ*g* vs 1/*M*. This value could be used to correct for the underestimated *D* values in badly stirred solution according to eq 8:
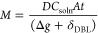
8

### Binding Capacity of the DGT Sampler

The effect of different environmental matrices on the binding capacity of the DGT sampler was studied. DGT samplers were deployed in 3 L solutions of 0.01 M NaNO_3_ or artificial seawater and Cs-133 concentrations range from 1 × 10^–6^ to 1 M for 25 h. Solutions were spiked with the Cs-137 tracer for the measurement of the accumulated Cs using the tracer technique and isotopic ratio according to the analytical procedures in the next section. The capacity was deduced from the plateau of a logarithmic plot of the molar concentration of Cs^+^ in solution vs the accumulated Cs^+^ mass on the DGT binding gel.

### Long-Term Deployment of DGT Samplers in Artificial Seawater

Artificial seawater was prepared according to the procedure described in the Supporting Information at pH 8.5 and then spiked with 10 Bq·L^–1^ Cs-137. Table S1 summarizes its ionic composition. To evaluate the DGT performance in seawater at the long-term scale of time, DGT samplers assembled with APA pretreated in 0.4 M NaCl for 3 days or AGE diffusive gels were deployed in a tank of 6 L. Samplers were retrieved after 1, 2, 3, and 4 week time intervals to measure the accumulated Cs-137. The tank was monitored every 2 days and compensated for Cs-137 depletion. The temperature was monitored every half an hour using a temperature logger. The average temperature for each interval was used for *D* corrections using eq 5. *C*_DGT_ was calculated using eq 6.

### Analytical Procedures

Cs-137 activity was measured using a high-purity germanium (HPGe) detector. Liquid samples were measured in 20 mL vial geometry. As for DGT binding phase disks loaded with Cs-137, a nondestructive procedure was adopted. They were individually spread on 25 mm paper disks, dried under an infrared lamp stored in self-sealing plastic bags, and then counted in the disk geometry for better efficiency compared to the cylindrical one associated with the conventional Cs-137 analysis methods. This is attributed to the decreased sample self-attenuation because of the negligible sample thickness and the solid angle offered by this geometry in a coaxial arrangement with the detector. Figure S1 shows the dried binding gel and the measurement arrangement used. For experiments involving the Cs-133 carrier, known amounts of Cs-137 tracer were added to the solution to calculate the Cs-133 concentration using the tracer technique.

### Precision, Accuracy, DGT Blanks, and Detection Limits

The precision of DGT measurements is an indication of the closeness and consistency of the replicate measurements to each other. It was quantified by the relative standard deviation (RSD) using eq 9:
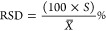
9where *S* is the standard deviation and *X* ® is the sample mean. The accuracy is a measure of how near the measured *C*_DGT_ is to *C*_soln_. It was expressed by the relative error (*E*_r_) according to eq 10:
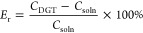
10

In this study, the acceptable limit for both *E*_r_ and RSD was 15%, which is similar to the limit accepted by Gorny et al.^15^ DGT blank measurements in mass were obtained by keeping triplicate samplers in a plastic bag for a month, retrieving the binding phase, and then making measurements according to the previously mentioned procedure. The DGT detection limit was calculated based on the mass (3 times the SD) and then using the DGT equation assuming deployment time and temperature to obtain a detection limit in concentration can be measured by DGT.

## Results and Discussion

### Uptake Kinetics of the Binding Phase

**Figure 1:**
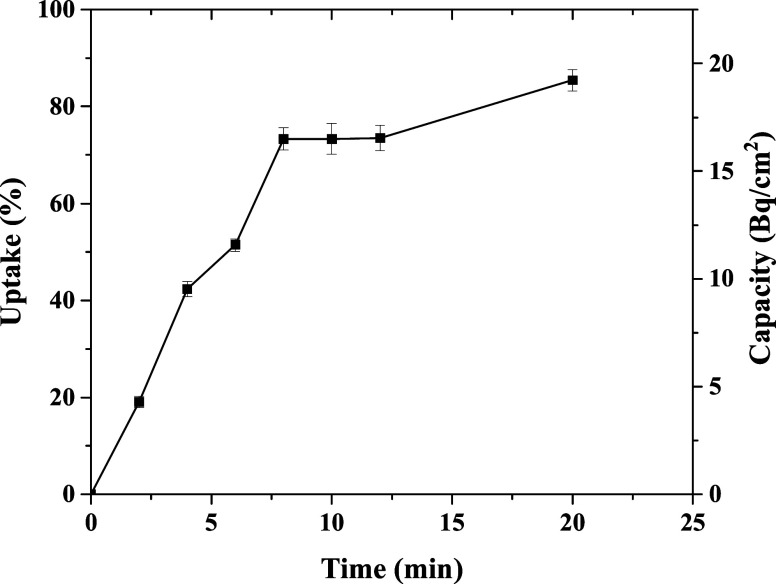
Cs-137 batch uptake kinetics in terms of percent uptake (%) and gel disk capacity (Bq·cm^–2^) of KZFCN binding gel from well-shaken 20 mL solutions of 6.1 Bq·mL^–1^ Cs-137 in 0.01 NaNO_3_.

The activity of ^137^Cs^+^ bound to the KZFCN binding gel disk sharply increased in the first 8 min. After that, a slow increase was observed, as shown in Figure 1. The binding rate after the first 4 min was 0.0192 Bq·s^–1^·cm^–2^, which was much greater than DGT fluxes (0.0011 Bq·s^–1^·cm^–2^) calculated from the DGT equation using the Cs^+^ diffusion coefficient, a diffusive layer of 0.094 cm, and a deployment solution of the same activity concentration at 25 °C. This illustrates that the rate of Cs-137 uptake by the KZFCN binding phase is fast enough to satisfy the DGT sampler theoretical demand, which suggests that the KZFCN binding phase is suitable for the DGT technique.

### Elution Efficiency

The elution of Cs^+^ from KZFCN binding gel disks was after equilibration with 100 μg·L^–1^ solution of Cs^+^ was tested with different eluents at different concentrations. The results are listed in Table 1. Nitric acid showed the lowest efficiency even at a concentration of 2 M. Ammonia exhibited a higher elution efficiency at a concentration of 1 M. This is due to the reaction of ferrocyanides and ammonia, which decomposes the binding phase.^22^ The addition of EDTA did not greatly improve the elution efficiency of the 1 M ammonia solution. The highest elution efficiency of 98.5% was obtained by using 1 mL of 2 M ammonia solution, which is lower than the ammonia concentration used for CFCN elution.^16^ Therefore, 1 mL of a 2 M ammonia solution was used for eluting Cs for this work.

**Table 1:** KZFCN Elution Efficiency (%) for Cs^+^ in Different Eluents at Various Concentrations

eluent	elution efficiency
1 mL 1 M HNO_3_	14.32 ± 2.69
1 mL 2 M HNO_3_	41.15 ± 4.53
1 mL 1 M NH_4_OH	71.98 ± 3.14
1 mL 1 M NH_4_OH + 25 mM EDTA	77.11 ± 5.09
1 mL 2 M NH_4_OH	98.46 ± 3.99

### DGT Exposure Experiment

**Figure 2:**
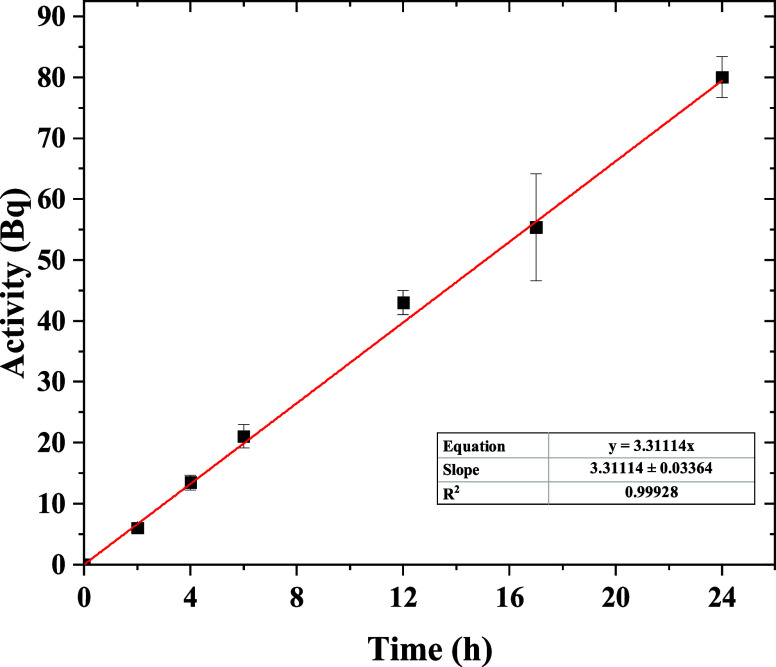
^137^Cs^+^ accumulation on the binding layer of the assembled DGT sampler vs time from 0.01 M NaNO_3_ solution containing 1.93 Bq·mL^–1^ Cs-137 (0.8 mm APA diffusive layer, pH = 7). Error bars represent SD.

Carrier-free Cs-137 was used for this experiment because it allows the direct measurement of the binding gel using an HPGe detector without further elution. DGT samplers were deployed in 0.01 M NaNO_3_ solution for different time intervals. The measured activity of Cs-137 in the binding gel increased linearly (*R*^2^ = 0.999) with deployment time (Figure 2). Both the fitted experimental data and the calculated results according to the DGT theoretical equation concurred. Consequently, the derived diffusion coefficient corrected for 25 °C agreed with the value measured by Chang et al. (1.92 × 10^–05^ cm^2^·s^–1^)^13^ using the diffusion cell method.

### Effects of pH and Ionic Strength on Gel Binding and DGT Performance

**Figure 3:**
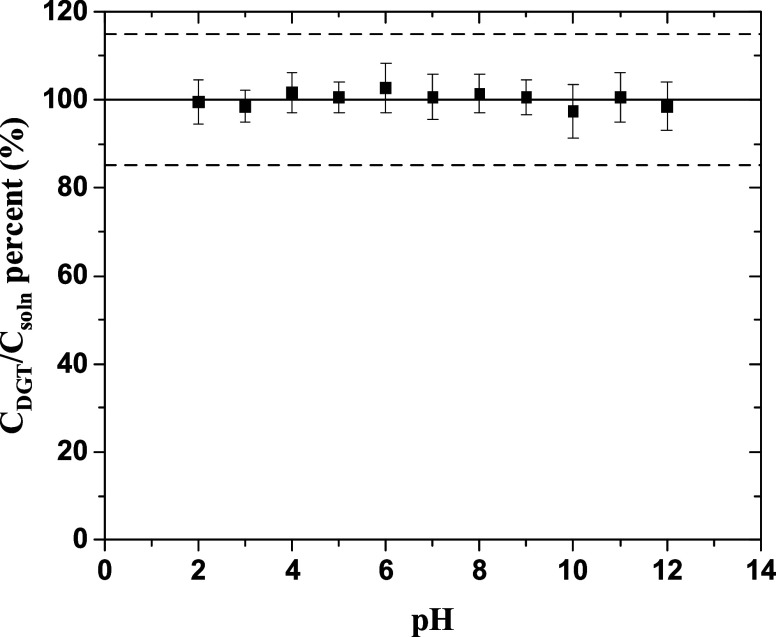
Effect of pH on the measured Cs-137 values using DGT, *C*_soln_ = 2.4 Bq·mL^–1^ in 0.01 M NaNO_3_, and exposure time = 24 h. Error bars represent the RSD. Acceptable *E*_r_ is outlined by dashed lines.

The Cs-137 batch uptake percentage by the KZFCN binding gel was unaffected by the solution pH (Figure S2). More than 95% of ^137^Cs^+^ activity was bound by KZFCN gel within the pH range of 2–12, which is wider than the range for natural waters. This range is broader than that for the previously studied binding gels: AG50W-X8,^13^ AMP,^14,15^ and CFCN.^16^ This is due to the higher stability of KZFCN over a wide range of pH compared to CFCN^23^ and AMP, which decompose in basic medium. The KZFCN used in this study has a lower tendency for decomposition, and therefore, cesium uptake is not affected at pH 12 in contrast to CFCN. The high percent of ^137^Cs^+^ uptake (97.3%) at pH 2 demonstrated higher selectivity of KZFCN for Cs^+^ over H^+^ ion compared to AG50W-X8, CFCN, and AMP binding phases. Consequently, the DGT technique based on the KZFCN binding gel could be used for the whole range of pH of natural waters, which is a significant improvement to the other previously studied binding phases. This was confirmed by DGT measurements in the same pH range. Figure 3 shows that the measured *C*_DGT_ values were within 95% of *C*_soln_ with acceptable RSD values.

**Figure 4:**
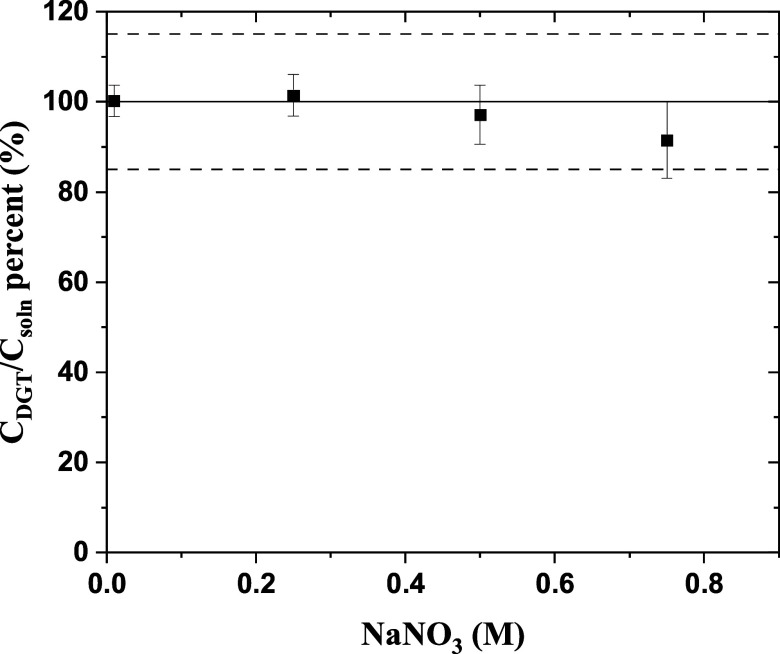
Effect of ionic strength on the measured Cs-137 values using DGT, *C*_soln_ = 2.4 Bq·mL^–1^, exposure time = 24 h. Error bars represent RSD. Acceptable *E*_r_ is outlined by dashed lines.

The cesium batch uptake by the binding gel decreased with increasing ionic strength, as shown in Figure S3. It was reduced by about 30% at the ionic strength near the seawater ionic strength (0.75 M NaNO_3_), compared to the uptake value at 0.01 M NaNO_3_. This is most likely due to the competition between Na^+^ ions and Cs^+^ for binding sites. However, the uptake percent is still satisfactory to meet the Cs^+^ flux supply in the range of natural waters. This was supported by DGT measurements in a series of solutions with NaNO_3_ concentrations of up to 0.75 M (Figure 4). The *C*_DGT_ decreased to 91.5% of the solution activity concentration with an acceptable RSD of 8.5%. However, it is still within the acceptable error range of DGT.

### Effect of Competing Ions on DGT Performance

**Figure 5:**
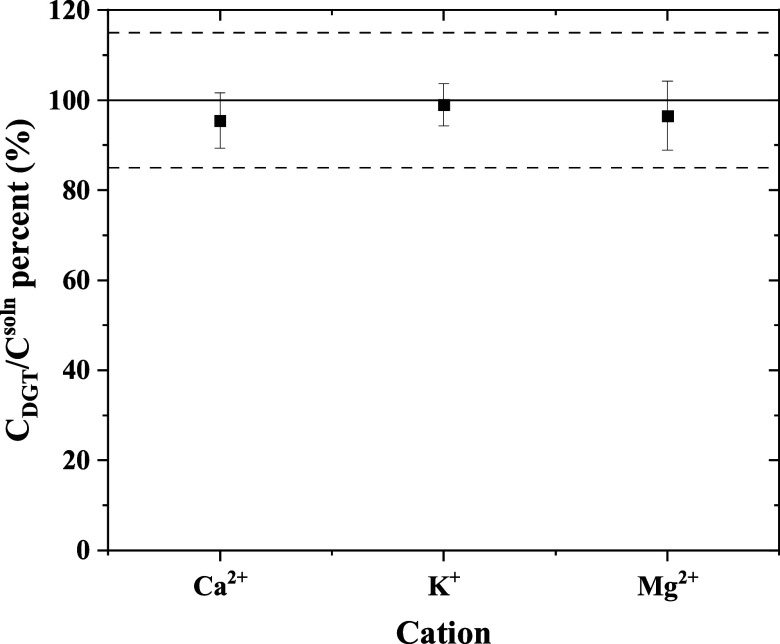
Effect of Ca^2+^, K^+^, or Mg^2+^ concentration on the DGT measurements expressed by the percentage of DGT-measured values to the solution concentration. Solutions compositions are 2.4 Bq·mL^–1^ Cs-137 in 300 mg·L^–1^ CaCl_2_, KCl, or MgCl_2_ and exposure time = 24 h. Error bars represent RSD. Acceptable *E*_r_ is outlined by dashed lines.

The presence of high concentrations of competing ions for Cs^+^ like Na^+^, K^+^, Ca^2+^, and Mg^2+^ in natural waters may limit the deployment time of DGT samplers. When the AG50W-X8-DGT sampler was deployed in soft water, its performance deviated after 25 h.^13^ AMP-DGT samplers were deployed for 18 h in Volvic water spiked with Cs^+^ and enhanced with 100 mg·L^–1^ Na+, Ca^2+^, or Mg^2+^. The DGT-measured concentrations of Cs^+^ were lower than the solution concentration by more than 15%.^15^ CFCN-DGT samplers showed a good performance in synthetic river water for over 14 days^16^ but were never deployed in seawater. In order to test the effect of competing ions on the DGT performance, 24 h DGT measurements in 300 mg·L^–1^ solutions of Ca^2+^, Mg^2+^, or K^+^ spiked with 2.4 Bq·mL^–1^ Cs-137 (Figure 5) were adopted. DGT response was not affected by any of those ions, which makes it a good candidate for seawater deployments.

### DGT Performance in Seawater

**Figure 6:**
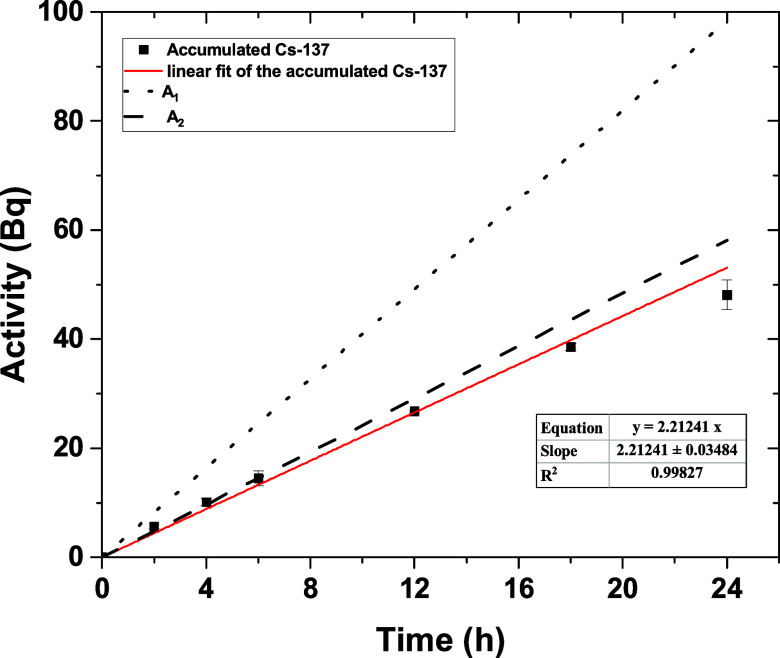
^137^Cs^+^ accumulation on the binding layer of the assembled DGT sampler vs time from artificial seawater spiked with 2.4 Bq·mL^–1^ Cs-137 (0.8 mm diffusive layer, pH = 8.5). Dotted line (A_1_) represents the calculated accumulated Cs-137 using the corrected D at 15 °C and without considering the DBL. Dashed line (A_2_) corresponds to the calculated accumulated Cs-137 using the corrected D at 15 °C and considering the DBL.

To evaluate the performance and applicability of KZFCN-based DGT samplers in seawater, they were initially deployed in artificial seawater spiked with 2.4 Bq·mL^–1 137^Cs^+^ for time intervals up to 24 h. The results represented in Figure 6 show the linear accumulation (*R*^2^ = 0.998) of Cs-137 in the DGT samplers over the period of deployment. The effective diffusion coefficient (*D*_eff_) of ^137^Cs^+^ in this experimental setup was 1.012 × 10^–5^ cm^2^·s^–1^ (corrected to 25 °C), which is lower than the value obtained in the previous section by 47%. This was illustrated by comparing the linear fitting of the measured data points (Figure 6, solid line) and the theoretical line (Figure 6, dotted line A_1_) generated using the *D* value obtained in the previous section. One of the possible reasons for lower *D*_eff_ values could be DBL.

The DBL thickness (δ_DBL_) was measured as described in the Materials and Methods section, and the results are shown in Figure S4. It illustrates a linear relationship (*R*^2^ = 0.999) between the reciprocal of accumulated Cs-137 and Δ*g*. The measured δ_DBL_ was 0.66 mm, which points out that the deployment solution was not well stirred enough to diminish the DBL. After adding the δ_DBL_ to the diffusion layer thickness, the calculated diffusion coefficient is 1.71 × 10^–5^ cm^2^·s^–1^ at 25 °C (Figure 6, dashed line A_2_), which is 10.7% lower than the measured values in 0.01 M NaNO_3_. The same was found with the diffusion coefficients of other ions in seawater, which were about 9% less than those measured in solutions without any electrolytes.^24^ This also justifies the lower *C*_DGT_ value measured in Figure S3 at an ionic strength of 0.75 M.

### Binding Capacity of DGT

**Figure 7:**
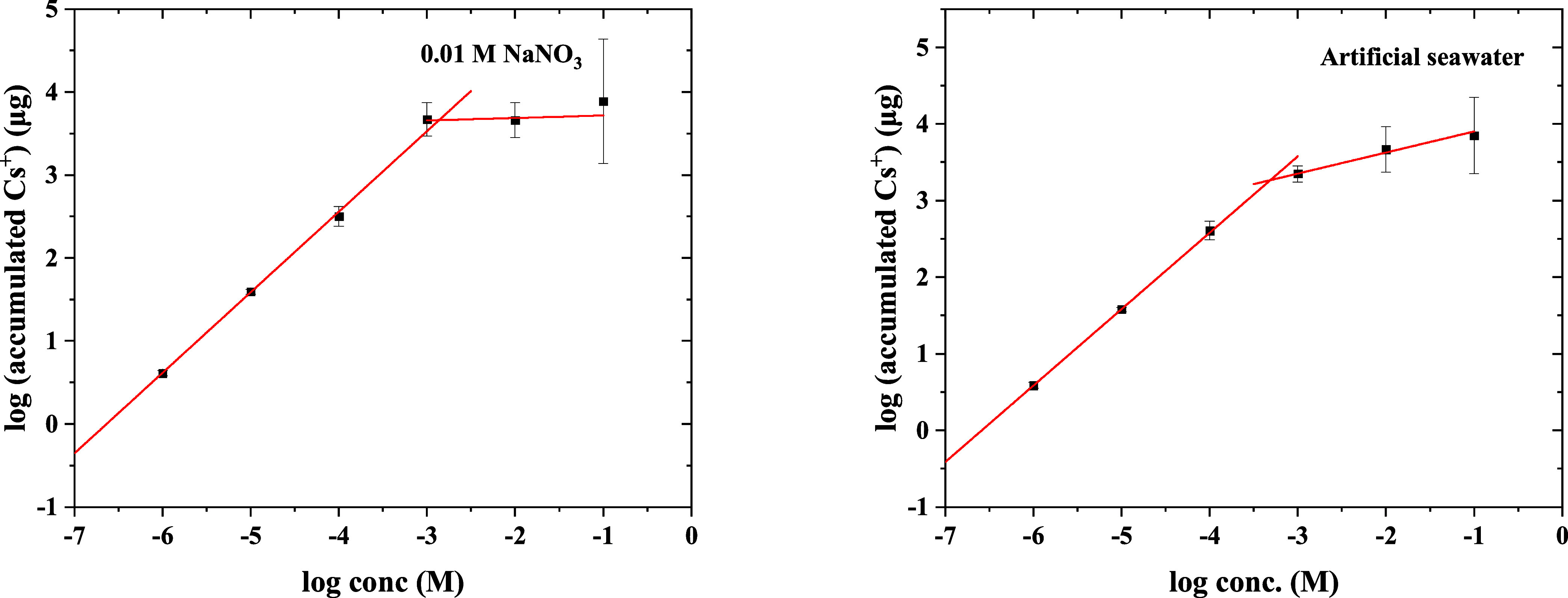
Accumulated Cs^+^ mass on the DGT samplers deployed in a range of Cs^+^ concentrations in 0.01 M NaNO_3_ (at the left) and artificial seawater (at the right) for 25 h.

The capacity of the binding phase is one of the factors that has an effect on the length of the deployment time. The results shown in Figure 7 illustrate the linear increase of the accumulated Cs^+^ by increasing the solution concentration. The plateau on the graphs suggests that the binding capacity of the DGT sampler was reached. The maximum capacity in 0.01 M NaNO_3_ was 4.54 mg per single DGT sampler. This value corresponds to 428.62 mg of Cs^+^ per 1 g of KZFCN, which is close to reported values for KZFCN (538.5 mg/g^25^). Capacity measurement in seawater medium exhibited a significant decrease to 1.84 mg per DGT unit. This could be attributed to the increased competition between Cs^+^ and the dissolved cations, which saturate the KZFCN adsorption sites, and only the highly Cs specific sites become available for Cs^+^ adsorption.^26^ Considering the natural Cs-133 concentration of 0.4 μg·L^–1^ in seawater and a temperature of 10 °C, the KZFCN-DGT sampler can be theoretically deployed for about 194.7 years until the sampler reaches 50% of the saturation.

**Figure 8:**
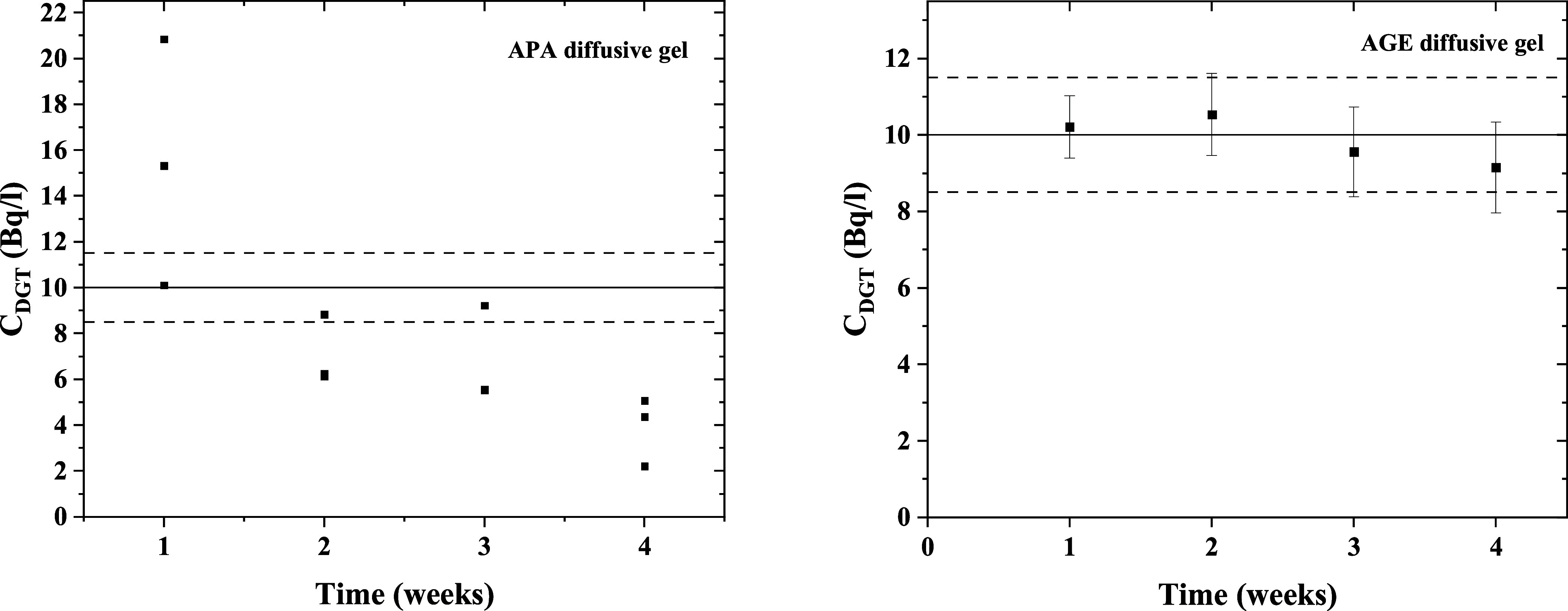
DGT-measured specific activity of Cs-137 in artificial seawater solution spiked with carrier-free 10 Bq·L^–1^ Cs-137 using APA (left) or AGE (right) as diffusive gels.

Long-Term Deployment of DGT Samplers in Artificial Seawater. To further assess if the KZFCN-DGT deployment time is limited by the major ion competition or not, samplers equipped with APA diffusive gels were deployed for 4 weeks in artificial seawater spiked with 10 Bq·L^–1^ Cs-137. The experimental details are given in the Materials and Methods section. The DGT-measured activity concentrations were weekly monitored and compared to the solution activity concentration (Figure 8). Although the results exhibited wide variations for the same period of deployment, which made them statistically inaccurate and imprecise, some measurements were within the *E*_r_ range of less than 15% *E*_r_. Examination of the samplers during retrieval of the gels revealed the shrinkage of the APA diffusive gels thicknesses to about 0.6 mm. As a result, the gels were not tightly held by the cap in most of the samplers. To confirm if the gel shrinkage effect was the reason for the statistically imprecise results, the same experiment was repeated using AGE diffusive gel, which is known for its shrinkage resistivity in different environmental matrices. With the AGE diffusive gel, the DGT-measured activity concentrations were all within the range of 8.5% *E*_r_ with an RSD of 13% at the 4th week. It can be concluded that AGE is more suitable for long-term deployments in seawater compared to 0.8 mm APA diffusive gel. On the other hand, the KZFCN binding phase showed a high selectivity to Cs-137 and its performance was not limited by the presence of high concentrations of competing ions like the other binding phases, such as AMP and AG50W-X8.

### Detection Limit

γ spectrometric counting using an HPGe detector of blanks revealed no Cs-137 activity. More formally, values were below the detection limit, which points out that the KZFCN-DGT samplers can be used for the measurement of low-level Cs-137 activity. Consequently, the DGT detection limit is a factor of the deployment time and the detector detection limit. Assuming an HPGe detector detection limit of 10 mBq and a DGT sampler of 0.8 mm diffusive gel with a 0.14 mm membrane filter and a window area of 3.14 cm^2^ is deployed for 30 days at a temperature of 15 °C, the DGT detection limit would be 4 mBq·L^–1^. A better detection limit could be achieved by increasing the deployment time or exposure window area or decreasing the diffusive gel layer thickness. This detection limit is enough for the measurement of low-level Cs-137 activity at the oceanic background level, which is in the range of few mBq·L^–1^.^27^

## Conclusions

The performance of the KZFCN binding phase-based DGT sampler for the measurement of Cs-137 in seawater was investigated. The KZFCN binding gel behavior in various conditions was evaluated through batch and time-series DGT experiments. The gel was approved as a fast and strong binding phase for Cs-137 in the pH and salinity ranges of natural waters. A time series of KZFCN-DGT in artificial seawater for 24 h deployment showed that the diffusion coefficient is 10.7% less than that measured in 0.01 M NaNO_3_, which is reasonable and in agreement with literature values. The results of 4 week deployments of the KZFCN-DGT sampler in seawater indicated that the Cs measurements were not affected by the ion competition in seawater. AGE diffusive gels showed a better performance in seawater as APA diffusive gel has a tendency for shrinkage. Preconditioning APA sheets in seawater before cutting may reduce shrinkage. The studies on capacity have revealed that it was affected by strong competition of other ions in seawater but was still high enough for long-term deployment and for making accurate measurements. The coupling of γ spectrometry with the developed DGT technique eliminated the elution step, making it more robust and easy to use. The appropriate detection limit was established for measuring low-activity concentrations of Cs-137 in seawater, and it can be further improved by increasing the deployment time or/and reducing the thickness of the diffusion layer. The developed KZFCN-DGT technique can be applied for studying the Cs bioavailability and geochemical processes in aquatic systems.

## Supporting Information Available

Artificial seawater preparation method and a table of its ionic composition, table of *D*_Cs^+^_ at different temperatures in seawater and water, photographs for Cs-137 in the binding phase measurement setup on the HPGe detector, graphs for the pH and ionic strength effect on Cs-137 uptake by the binding gel and the δ_DBL_ measurement. (PDF)
