# Supplementary material for: Development of a Novel DGT Passive Sampler for Measuring Cs-137 In Situ in Marine Environments
Source: Anal Chem. 2024 Feb 13;96(8):3300–7. doi: 10.1021/acs.analchem.3c03767 (PMC10902808; doi:10.1021/acs.analchem.3c03767)
Supplement: Supplementary file 1 — ac3c03767_si_001.pdf [file ac3c03767_si_001.pdf]

Supporting Information

Development of a novel DGT passive sampler for measuring Cs-137 in situ in marine environments

Ahmed Elsenbawy[a,b*], Jacqueline M. Pates[b], Nariman H. M. Kamel[a], Tarek Morsi[a], Mohammed Mekewi[c], Ayman El-gamal[d], Nabawia A. Moussa[c], Hao Zhang[b]

[a] Radiation Protection Department, Nuclear Research Center, Egyptian Atomic Energy Authority, Cairo, 13759, Egypt

[b] Lancaster Environment Centre, Lancaster University, Lancaster, LA1 4YQ, United Kingdom

[c] Department of chemistry, Faculty of Science, Ain Shams University, Cairo, 11566, Egypt

[d] Marine Geology Department, Coastal Research Institute, National Water Research Center, 15, St. Elpharanaa, Elshalalat, Alexandria, 21514, Egypt

[*]E-mail address: a.elsenbawy@lancaster.ac.uk

Contents

Materials and methods: Artificial seawater preparation.

Table S1. Ionic Composition of ASTM D1141-98 seawater.

Table S2. Cs^+ Diffusion coefficients (D) in water and artificial seawater (ASTM D 1141-98) (40.9 $g.Kg^{-1}$) at different temperatures.

Figure S1. Dried binding phase on 25 mm paper disk (left) for the direct measurement of Cs-137 by HPGe and the geometry set up on the detector (right).

Figure S2. Effect of pH on the batch percent uptake of Cs-137 by KZFCN binding gel from well shaken 20 ml solutions of 6.1 $Bq.mL^{-1}$ Cs-137 in 0.01 $NaNO_3$.

Figure S3. Effect of ionic strength expressed by $NaNO_3$ concentration on the percent batch uptake of Cs-137 by KZFCN binding gel from well shaken 20 ml solutions of 6.1 $Bq.mL^{-1}$ Cs-137 for 24 h.

Figure S4. Effect of diffusive layer thickness on the accumulated $137Cs^+$ by DGT units exposed for 12 h in synthetic seawater spiked with 2.4 $Bq.mL^{-1}$ of $^{137}Cs^+$.

Artificial seawater preparation: Artificial seawater was formulated according to ASTM D1141 – 98 standard method [1]. To prepare 1 L of seawater, the following chemicals were mixed: NaCl (Sigma) (24.53g), MgCl2 (Sigma) (5.20g), Na2SO4(Sigma) (4.09g), CaCl2(Fisher) (1.16g), KCl (Sigma) (0.695g), NaHCO3 (Sigma) (0.201g), KBr (Fisher) (0.101g), H3BO3(Fisher) (0.027g), SrCl2 (Fisher) (0.025g), NaF (Fisher) (0.003g), Distilled water (988.968g). The solution was stirred in open air overnight to equilibrate with air CO_2.

Table S1. Ionic composition of ASTM D1141-98 seawater

Ion	Concentration (mg.L^{-1})
Na^+	10368.62
K^+	397.59
Ca^{2+}	418.83
Mg^{2+}	1327.16
Sr^{2+}	1.37
Cl^-	19402.14
SO_4^{2-}	2764.29
HCO^{3-}	145.95
Br^-	67.81
F^-	1.36

Table S2. Cs^+ Diffusion coefficients (D) in water and artificial seawater (ASTM D 1141-98) (40.9 g.Kg^{-1}) at different temperatures

	Water	Seawater
**Viscosity (η) (mPa.s) at 15 ℃**	1.1375[2]	1.252[3]
**D (×10^{-5}) (cm^2.s^{-1}) at 15 ℃ (measured)**	1.45	1.32
**Viscosity (η) (mPa.s) at 25 ℃**	0.89[2]	0.970[3]
**D (×10^{-5}) (cm^2.s^{-1}) at 25 ℃ (calculated)**	1.92	1.71
**Viscosity (η) (mPa.s) at 21.09 ℃**	0.9775[2]	1.063[3]
**D (×10^{-5}) (cm^2.s^{-1}) at 21.09 ℃ (calculated)**	1.73	1.58

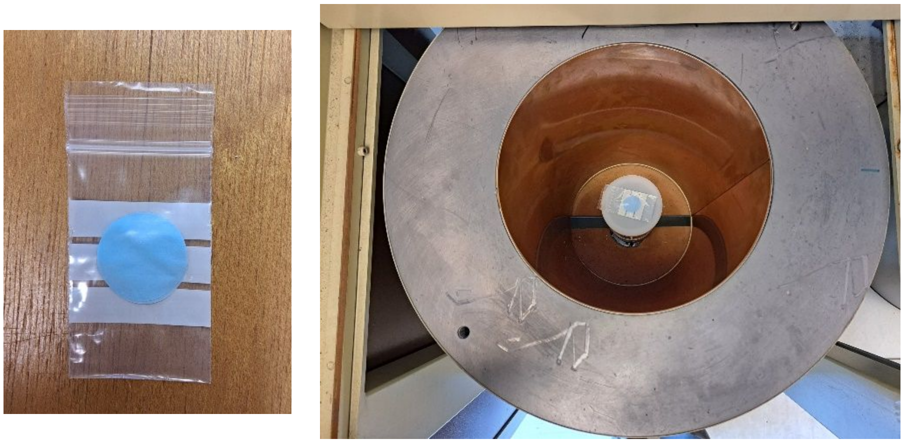

Figure S1. *Dried binding phase on 25 mm paper disk (left) for the direct measurement of Cs-137 by HPGe and the geometry set up on the HPGe detector (right).*

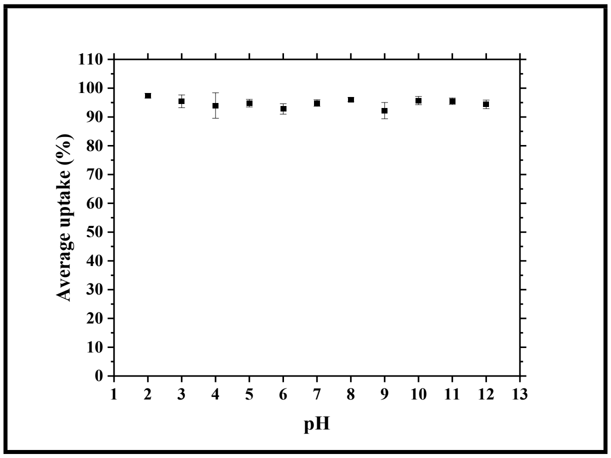

Figure S2. *Effect of pH on the batch percent uptake of Cs-137 by KZFCN binding gel from well shaken 20 ml solutions of 6.1 $Bq.mL^{-1}$ Cs-137 in 0.01 $NaNO_3$. Error bars represent SD.*

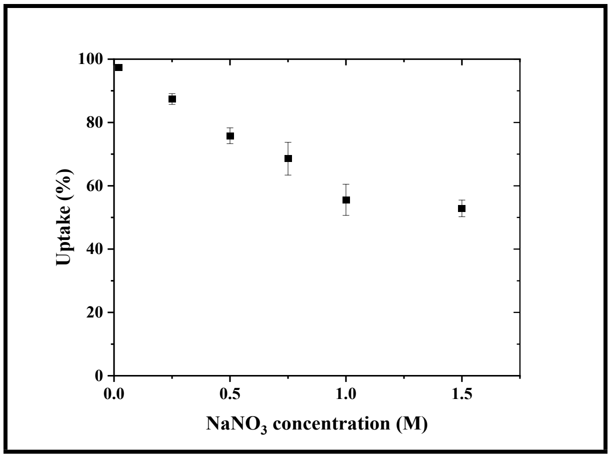

Figure S3. *Effect of ionic strength expressed by $NaNO_3$ concentration on the percent batch uptake of Cs-137 by KZFCN binding gel from well shaken 20 ml solutions of 6.1 Bq.mL^{-1} Cs-137 for 24 h. Error bars represent SD.*

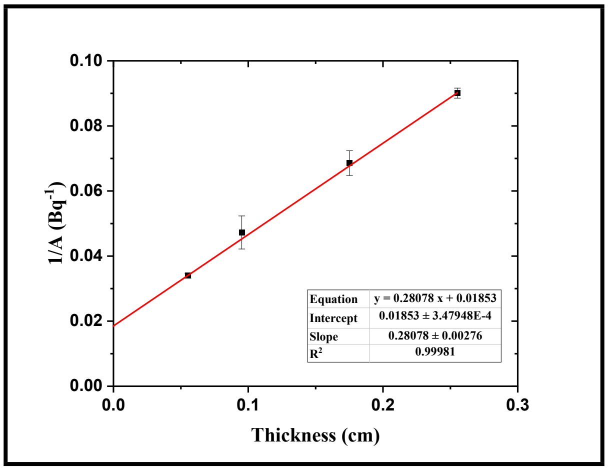

Figure S4. *Effect of diffusive layer thickness on the accumulated $^{137}Cs^{+}$ by DGT units exposed for 12 h in synthetic seawater spiked with 2.4 Bq.mL^{-1} of Cs-137. Error bars represent SD.*

■ References

(1) *ASTM D1141-98, Standard Practice for Preparation of Substitute Ocean Water*; 2021.

(2) IAPWS. *Release on the IAPWS Formulation 2008 for the Viscosity of Ordinary Water Substance*; 2008.

(3) Sharqawy, M. H.; Lienhard V, J. H.; Zubair, S. M. Thermophysical Properties of Seawater: A Review of Existing Correlations and Data. *Desalin. Water Treat.* **2010**, *16* (1–3), 354–380. https://doi.org/10.5004/DWT.2010.1079.
